# Supplementary material for: The Emericellipsins A–E from an Alkalophilic Fungus Emericellopsis alkalina Show Potent Activity against Multidrug-Resistant Pathogenic Fungi
Source: J Fungi (Basel). 2021 Feb 21;7(2):153. doi: 10.3390/jof7020153 (PMC7924852; doi:10.3390/jof7020153)
Supplement: Supplementary file 1 [file jof-07-00153-s001.pdf]

Supplementary

Table S1. Characterization of the clinical mold and yeast isolates from hospitalized patient in Moscow Government Health Department Scientific and Clinical Antituberculosis Center (MIC μg/mL), Sensititre YeastOne antifungal susceptibility test.

Strain ID	VOR	IZ	PZ	KZ	FZ	AmpB	AND	CAS	MF	5-FC
Aspergillus niger **1133m**	8	1	0,5	16	>256	1	>8	>16	>8	>64
A. fumigatus **390m**	0,5	0,25	0,25	8	>256	1	>8	>16	>8	>64
A. terreus **497**	0,25	0,25	0,12	8	>256	**R**	>8	>16	>8	>64
Candida albicans **1402**	1	0,5	0,5	4	256	0.5	0,06	0,06	0,03	0,06
C. glabrata **1402**	8	>16	>8	4 S	>256	1	0,06	0,06	0,015	0,06
C. krusei **1447**	1	0,5	0,5	0,5	**R**	2	0,03	0,25	0,12	32
C. tropicalis **156**	4	>16	>8	0,5	64	0.5	0,12	0,06	0,03	0,06
C. parapsilosis **571**	1	0,5	1	0,5	128	1	2	1	1	0,5
Cryptococcus neoformans **297**	0,25	0.5	0,25	0,06	16	1	>8	16	>8	8
Cr. laurentii **325m**	0,5	0,12	1	0,12	32	0.5	>8	16	>8	4

VOR - voriconazole, IZ - itraconazole, PZ - posaconazole, KZ – ketoconazole, FZ - fluconazole, AmpB - amphotericin B, AND - anidulafungin, CAS - caspofungin, MF - micafungin, 5-FC – flucytosine; R - intrinsic resistance.

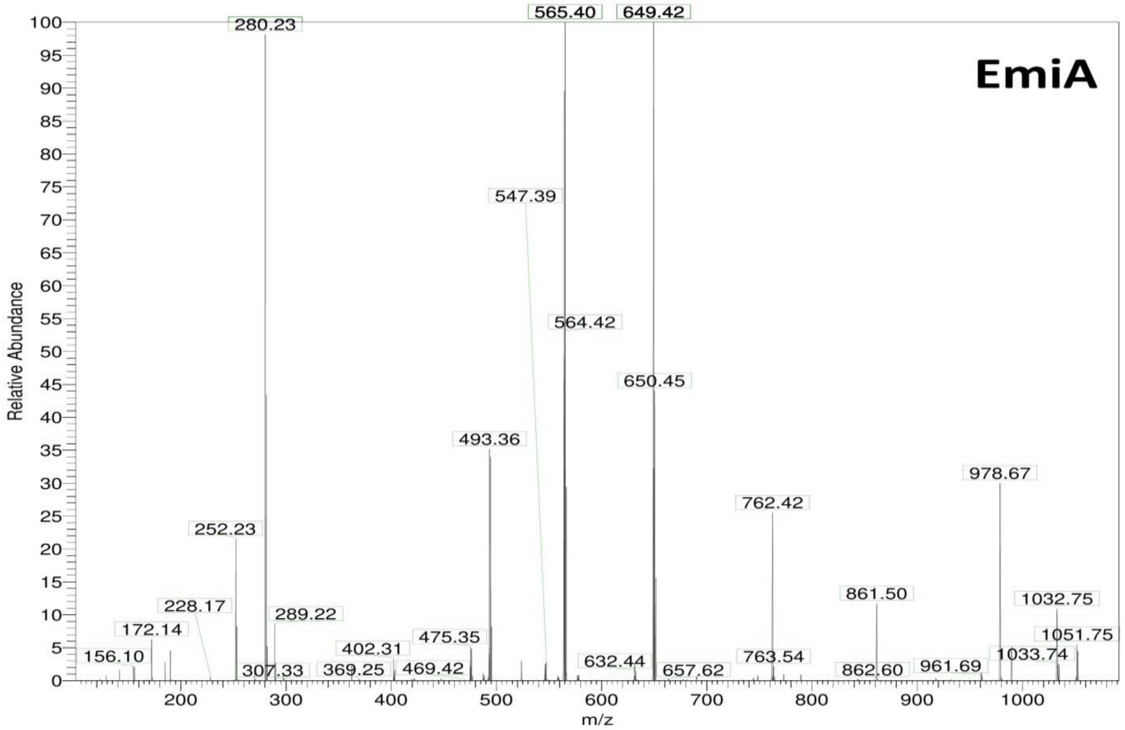
EmiA
Relative Abundance
m/z
280.23
565.40
649.42
547.39
564.42
650.45
493.36
978.67
762.42
252.23
861.50
1032.75
228.17
289.22
172.14
475.35
1051.75
402.31
156.10
632.44
763.54
1033.74
307.33
369.25
469.42
657.62
862.60
961.69

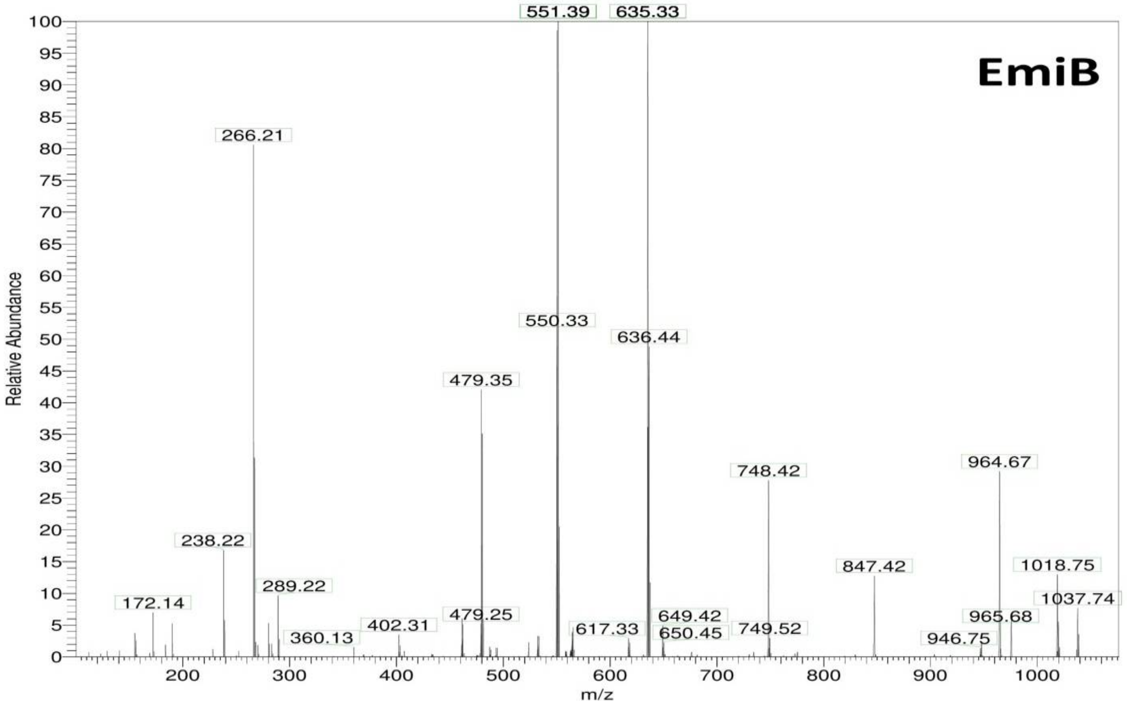
EmiB
Relative Abundance
m/z
551.39
635.33
266.21
550.33
636.44
479.35
748.42
964.67
238.22
847.42
1018.75
289.22
172.14
1037.74
402.31
479.25
649.42
965.68
360.13
617.33
650.45
749.52
946.75

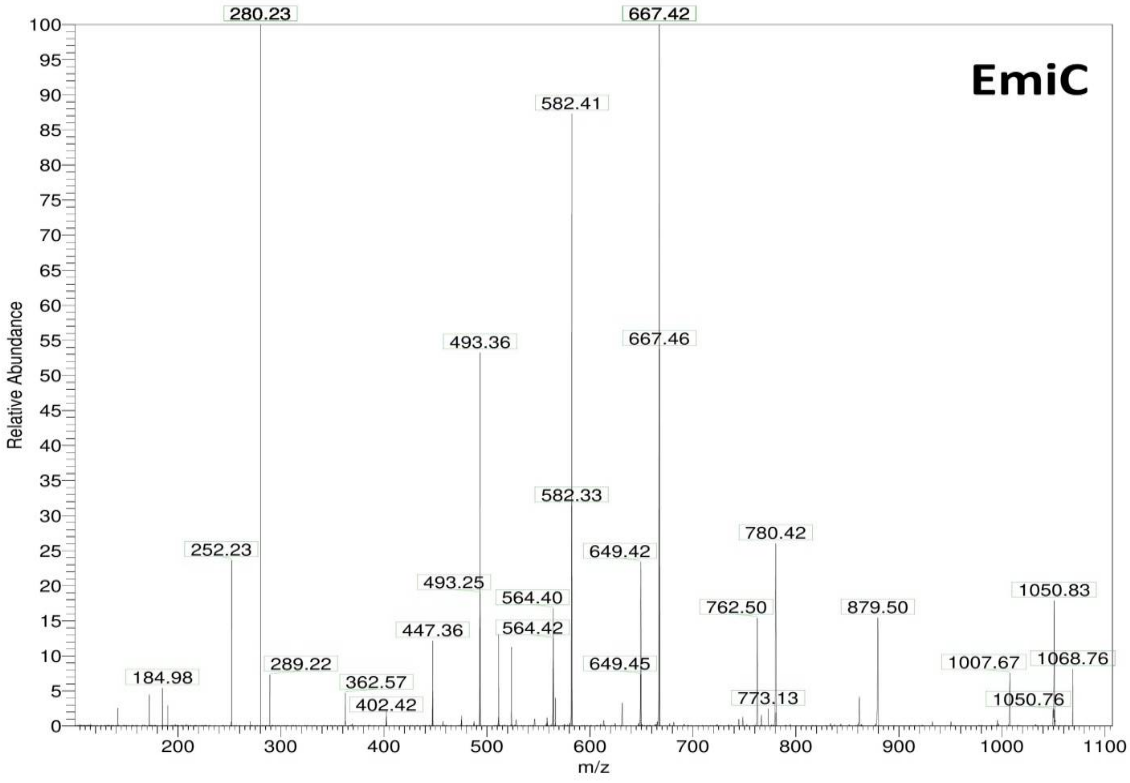
EmiC
Relative Abundance
m/z
280.23
667.42
582.41
493.36
667.46
582.33
252.23
649.42
780.42
493.25
564.40
762.50
879.50
1050.83
447.36
564.42
289.22
649.45
1007.67
1068.76
184.98
362.57
402.42
773.13
1050.76

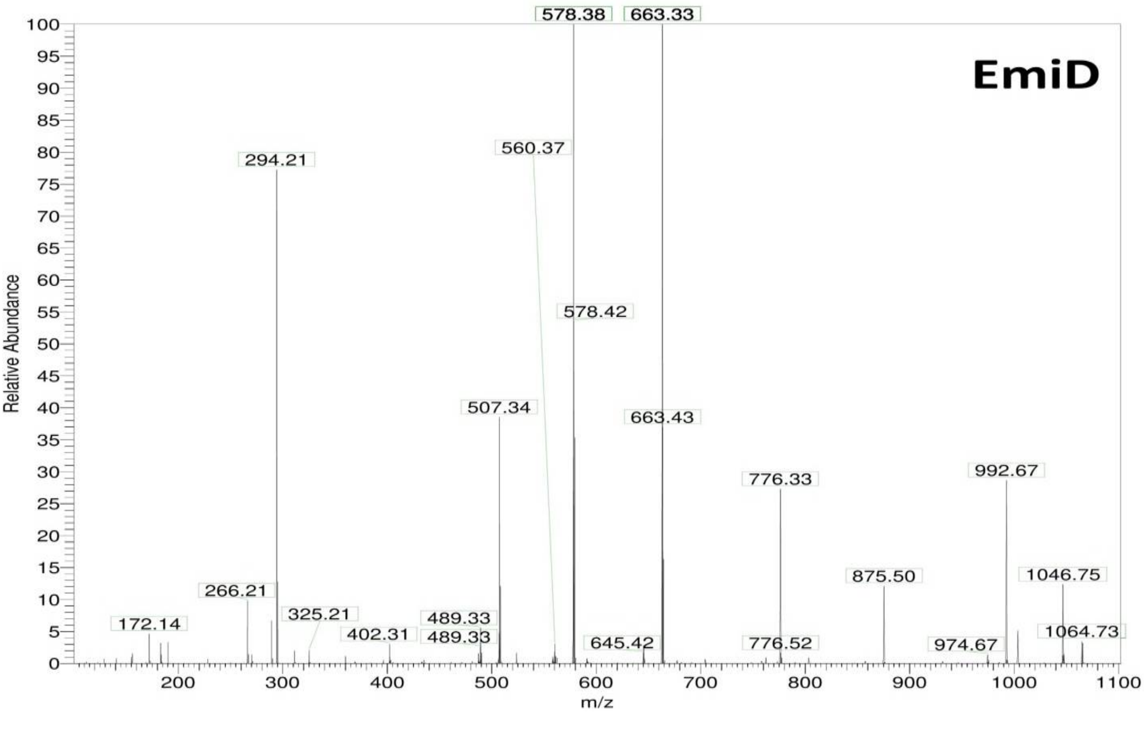
EmiD
Relative Abundance
m/z
578.38
663.33
294.21
560.37
578.42
507.34
663.43
776.33
992.67
875.50
1046.75
266.21
325.21
172.14
489.33
402.31
489.33
645.42
776.52
974.67
1064.73

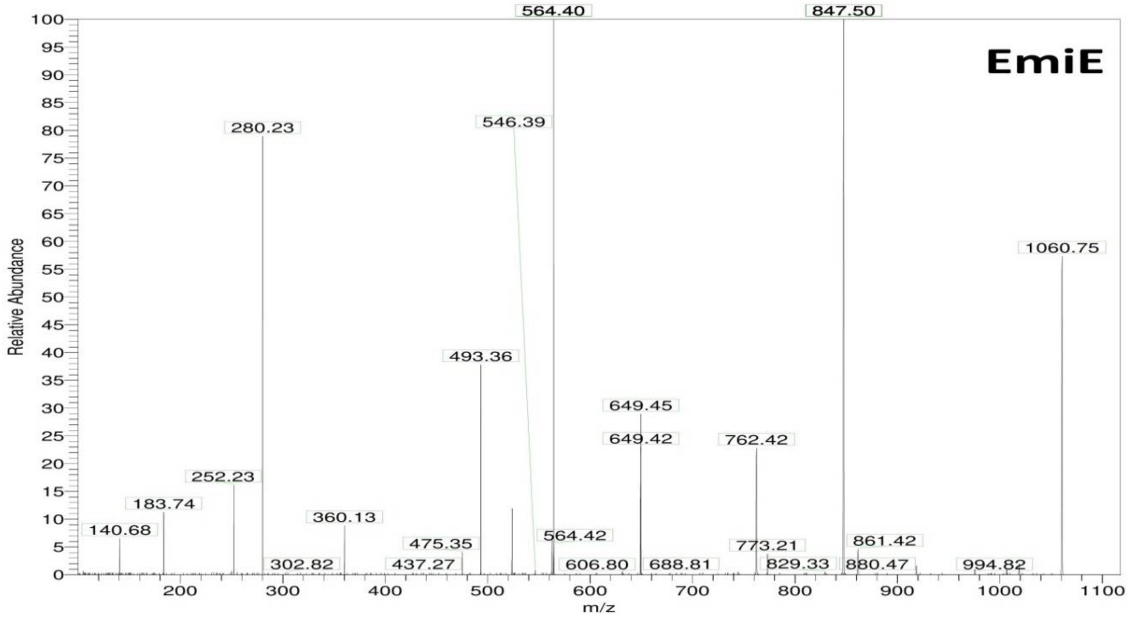

Figure S1. MS/MS analysis of the Emericellipsin A-E compounds.

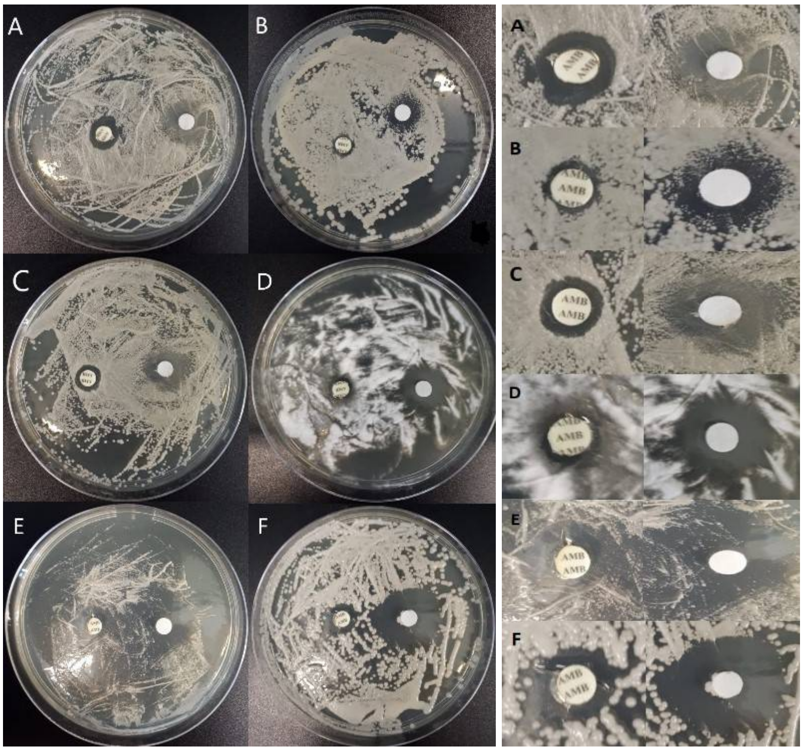

AmpB EmiA

Figure S2. Antifungal activity of EmiA and AmpB in disk diffusion assays against clinical pathogenic isolates: A - *Candida albicans* 1402, B - *Candida krusei* 1447, C - *Candida tropicalis* 156, D - *Aspergillus niger* 1133 m, E - *Aspergillus fumigatus* 390m, F - *Cryptococcus neoformans* 297

.

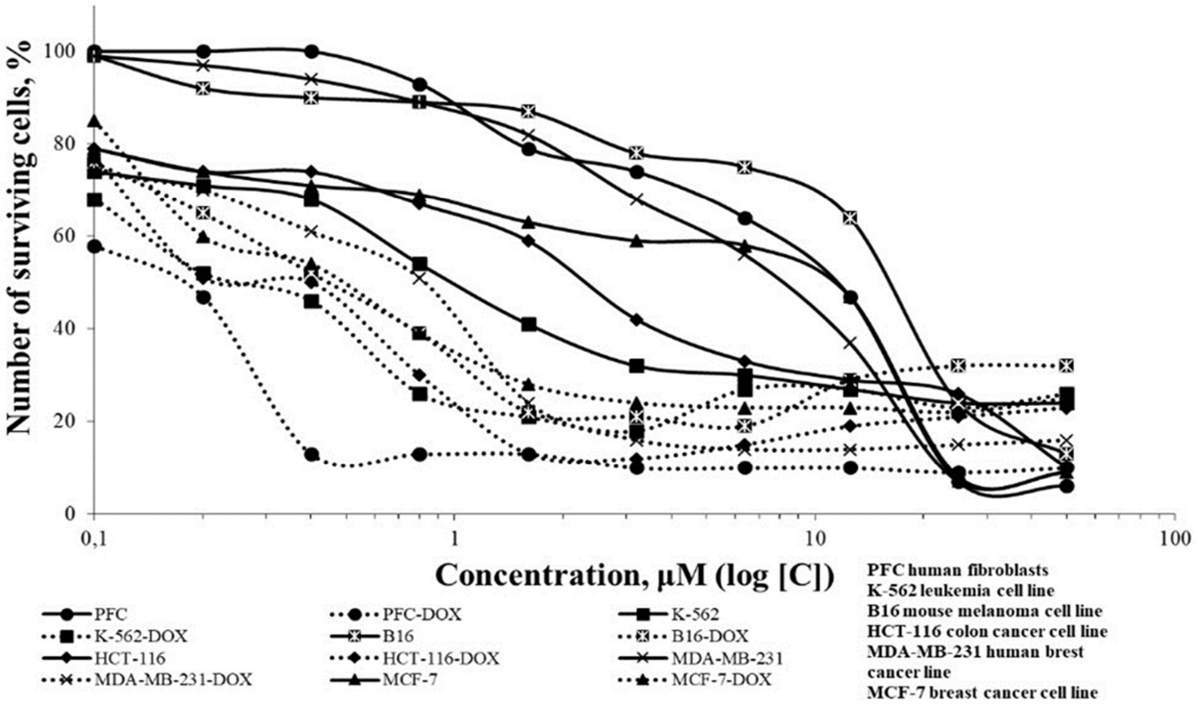

Figure S3. Cytotoxic activity caused by the EmiA peptaibol towards tumor and normal cell lines *in vitro.*

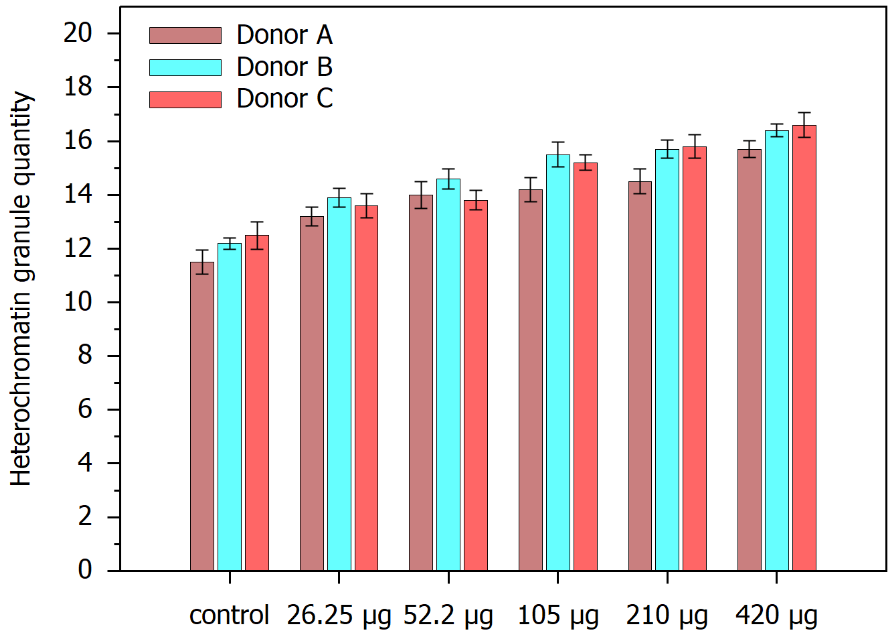

Figure S4. Heterochromatin condensation in human buccal epithelium cells caused by incubation with EmiA at different concentrations. Donors A, B and C represented by three different humans who provided a donor tissue.
